# The Medical Treatment of School Children

**Published:** 1909-03-27

**Authors:** A. D. Edwards

**Affiliations:** Medical Officer, Bournemouth Education Committee


### SPECIAL ARTICLE.

### THE MEDICAL TREATMENT OF SCHOOL CHILDREN: A SUGGESTION FOR INSURANCE.

By A. D. EDWARDS, M.B., B.S. (Lond.), B.Sc., D.P.H., L.R.C.S.; Medical Officer, Bournemouth Education Committee.

During the past few months, the publication of the reports of the recently appointed school medical officers has revealed a condition of things which has startled many people. In thirty per cent. of our elementary school children there exists a defect or ailment which is a handicap to healthy vigorous growth; and in many cases the ailment, if not cured, will have a serious effect in lowering the health standard of the future adult.

What this means to the individual in physical suffering, and to the State in the diminished value of its future citizens is difficult to realise, but there is one fact which gives us hope: that is that the majority of these defects and ailments can be cured if they are treated in the early stages.

### The Cost of Treatment.

Before I attempt to deal with the wider aspect of the question, it will be well to consider the extent to which the parents of these children are able to pay for the medical treatment of these conditions. A very large percentage of these ailments, nearly 70 per cent., is made up of:—

(1) Eye diseases and defective vision;
(2) Ear disease;
(3) Nose and throat disease;
(4) Carious teeth; and for none of these conditions is it possible for the parents to obtain adequate treatment at a small cost.

The charges for the treatment of eye diseases and vision testing, operations on the nose and throat, and the efficient treatment of ear disease are beyond the means of the average parent of the elementary school child; and with regard to dentistry, payment by these parents for "filling and stopping," which will leave the child with a set of teeth sufficient for the purposes of healthy digestion, is impossible; and dental treatment, if obtained at all, resolves itself into extraction.

The character and the thoroughness of the treatment required for the conditions mentioned, make it impossible for the majority of the parents to pay the cost. Some parents can afford to pay the whole cost, but they are few; most of the parents can afford to pay only a very small part of the cost, whilst some can afford to pay nothing.

It may possibly occur to some of my readers that the parents of these children, and indeed, the children themselves, may be members of medical clubs or provident associations; and this is true. But inasmuch as these organisations do not provide for vision testing, and operations on the ear, throat, and nose, their value in the present instance is correspondingly small; nor, under the present conditions, do they provide treatment, other than extraction, for carious teeth.

### The State, the Parent, and the Child.

The extent to which the State would benefit by the treatment of these children is great. Each defect and ailment cured means a more valuable future citizen: in many cases the cure means the difference between a weakly adult and a vigorous healthy being; and inasmuch as the prosperity of the State depends largely on the well-being of its citizens, the total advantage would be enormous. The natural conclusion of this line of argument is that it would be a good investment for the State to provide treatment for these children.

It would; but there is another factor to be considered—that of parental responsibility. Because it is an influence of the utmost value on the citizen, this responsibility should be fostered; whatever is done, it should be diminished as little as possible. When the parent *can* afford to pay for the treatment of his child's condition he should not shirk his responsibility. But it is clear that the treatment of these conditions is beyond the means of the average parent in question, so that we have on the one hand the opportunity of the State to improve and fortify its position, and on the other hand the inability of the parent to pay for treatment; if the State provides free treatment, it will do so at the risk of sacrificing parental responsibility; if the State does not provide free treatment, the children will be handicapped by their continued ailments and will grow up into citizens of diminished value, for the parents cannot afford the necessary treatment.

### A Scheme of Insurance.

It appears to me that it will be possible in the future to evolve a scheme whereby these difficulties will be obviated. In this scheme the parent would insure against the chance of his child developing these school-age ailments and defects; the premium would be within his means and might be made up of monthly payments; and because the State would benefit by the early treatment and cure of defects which the child might develop, it would be just that the State should contribute part of the premium. A parallel case may be found in the obligatory insurance of German workmen against sickness, the workman paying two-thirds and the employer one-third of the cost. As an alternative arrangement, the same system might be applied to a Provident Medical Association, each parent making a small monthly payment which would be supplemented by one-half its value by the State, the payment to continue during the school age of the child.

It may be suggested that such an arrangement might be grafted on to the club system which obtains at present throughout the country. But this would be inadvisable, for reasons to be stated, and a *sine quâ non* for efficiency in the treatment of these defects is the selection of the medical man by the parent, and payment from the central fund for each case treated and each operation performed.

The present system of medical club practice has brought many evils in its train; frequently it has resulted in the "sweating" of the medical attendant, and it has made neither for greater efficiency in medical treatment nor for the well-being of the patients. If some practical scheme of insurance could be evolved whereby the parent would contribute two-thirds and the State one-third of the cost, parental responsibility would not be interfered with, and the State would undertake its responsibility to the citizen and would be safeguarding its future. But such a scheme cannot be evolved in a day, and the condition of more than a third of our elementary school children cries out for treatment. We now know what is needed; the ailments and defects of thousands of children throughout the country are recorded, and these conditions are causing permanent damage to these children. So that whatever arrangements are made for the future, whatever schemes are evolved, it is necessary to deal with these children at once. Delay is dangerous; in a few years these conditions will be much less easily cured, they will have caused more permanent damage. Early treatment is essential, and if we are to await the evolution of a scheme, we shall be losing the opportunity of curing many thousands of our future citizens.

